# Hirschsprung’s disease associated with alopecia universalis congenita: a case report

**DOI:** 10.1186/s13256-016-1035-z

**Published:** 2016-09-15

**Authors:** Sushma Malik, Mani Singhal, Shruti Sudhir Jadhav, Charusheela Sujit Korday, Chitra Shivanand Nayak

**Affiliations:** 1 Division of Neonatology, Department of Pediatrics, T.N. Medical College & B.Y.L Nair Hospital, Block no 3, Flat no 7, Brady’s Flats, Sorab Bharucha Road, Colaba, Mumbai, 400005 India; 2 Department of Dermatology, T.N. Medical College & B.Y.L Nair Hospital, Mumbai, 400008 India

**Keywords:** Intestinal aganglionosis, Congenital atrichia, Hypotrichosis, Hirschsprung’s disease, Alopecia universalis congenita

## Abstract

**Background:**

Hirschsprung’s disease is one of the commonest causes of intestinal obstruction in neonates because of gut motility disorder. It is characterized as a complex genetic heterogenous disorder with variable inheritance. Hirschsprung’s disease occurs as an isolated phenotype in majority (70 %) of cases. In other cases it may be associated with syndromes (such as Down’s syndrome, Waardenburg syndrome, congenital central hypoventilation, or cartilage–hair hypoplasia) or with a spectrum of congenital anomalies involving neurological, cardiovascular, or urological systems or with sensorineural anomalies. In our patient, Hirschsprung’s disease was associated with alopecia universalis. Alopecia universalis congenita is a rare disorder of skin characterized by generalized absence of hair at or shortly after birth. The inheritance patterns range from autosomal recessive, dominant or X-linked recessive forms. The autosomal recessive form is the most common and severe type in which patients present with complete absence of hair development, affecting the entire scalp and body. Alopecia universalis congenita occurs either in isolation or as a part of congenital syndromes. Here, we report the case of a neonate who presented with Hirschsprung’s disease with alopecia universalis congenita, an association which has not been reported before.

**Case presentation:**

A preterm (33 weeks’ gestation) 1.4 kg Indian baby girl was born to a gravida two mother by caesarean section. At birth, clinical examination revealed total absence of scalp and body hair. On day 3, she had bilious vomiting and a barium study was suggestive of Hirschsprung’s disease. An exploratory laparotomy and intestinal biopsy report revealed aganglionic muscularis propria; a skin biopsy from her scalp was suggestive of alopecia universalis. Postoperatively, she died due to multiorgan failure. Her family history revealed that her elder sibling also had alopecia universalis and esophageal atresia. This child died on day twelve. Our patient’s clinical features and the biopsy reports confirmed our diagnosis of Hirschsprung’s disease with alopecia universalis congenita.

**Conclusions:**

A diagnosis of Hirschsprung’s disease should make treating clinicians actively investigate for any associated syndromes and anomalies. Alopecia is an unusual association with Hirschsprung’s disease. Alopecia universalis congenita is the most severe form of alopecia areata. Early diagnosis and classification is essential for appropriate and timely management of such cases.

## Background

Hirschsprung’s disease (HD), also called congenital megacolon or intestinal aganglionosis, is caused by disruption of normal migration of colonic ganglionic cells during 5th to 12th week of gestation. It most commonly involves the rectosigmoid region of the colon; the entire colon and small intestine are rarely affected [1]. Varying lengths of constricted distal colon cause failure to pass meconium in newborns or less commonly patients present with persistent constipation, progressive abdominal distension, poor feeding, and failure to thrive. Confirmation is made by rectal suction biopsy which shows hypertrophic nerve trunks and absence of ganglion cells in the myenteric and colonic submucosal plexus [2].

Alopecia universalis congenita (ALUNC) is one of the rare disorders of skin; it is characterized by generalized absence of hair at birth or shortly after birth. This disorder occurs either as an isolated phenotype or is associated with other defects. Inheritance of ALUNC has been documented as autosomal recessive in the majority of cases and a few cases were documented as either autosomal dominant or X-linked. Recessive forms are prone to have a more severe phenotype in which complete absence of hair development is observed, affecting the scalp and entire body; therefore, it has been named alopecia universalis [3]. We report a rare case of a newborn baby girl born at our institute who had HD with alopecia universalis. To the best of our knowledge, she is the first case to be reported with this association.

## Case presentation

**Fig. 1:**
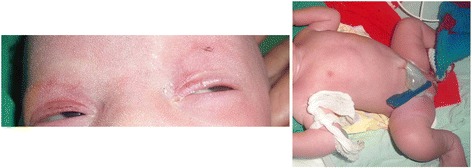
Alopecia universalis. No hair on entire body except for three to four strands of fine hair on the left eyebrow

**Fig. 2:**
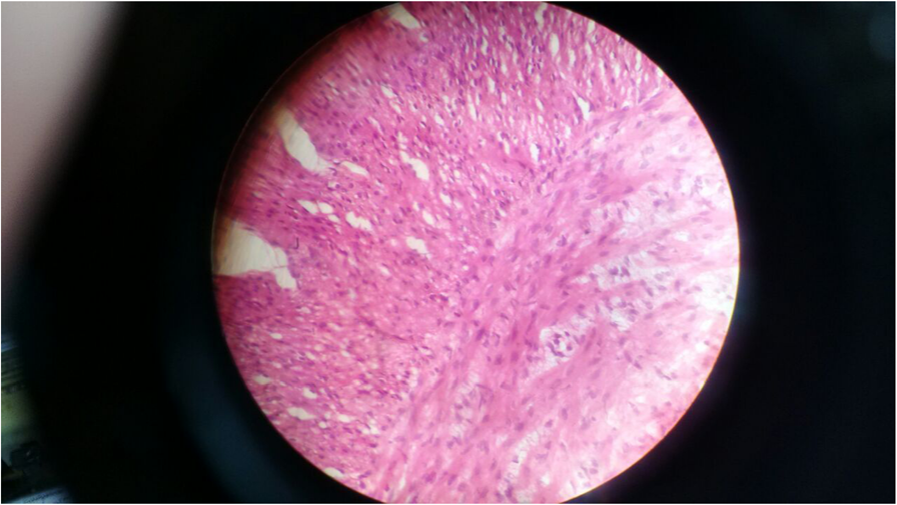
Biopsy from splenic flexure reveals no ganglion cells and presence of nerve bundles

A preterm (33 weeks’ gestation), 1.4 kg, small for gestational age, Indian baby girl was born to a second gravida mother by elective caesarean section done in view of oligohydramnios with obstructed labor. Her parents had a third degree consanguineous marriage. At birth, examination revealed total absence of scalp and body hair (except for three to four fine strands of hair on her left eyebrow; Fig. 1). No abnormality was detected in her skin or nails. There were no external congenital anomalies such as cleft palate, polydactyly, ear or eye abnormalities, skeletal malformations, or short stature. She had failed to pass meconium spontaneously for first 2 days and later passed only a small amount after rectal stimulation. On day three, she started having bilious vomiting and abdominal distension. Abdominal radiographs showed a dilated stomach and intestinal loops. A barium study revealed a long segment microcolon with transition zone at splenic flexure. An emergency exploratory laparotomy and ascending colostomy was done. Full thickness colonic biopsy samples were taken from her descending colon, splenic flexure, and stoma site. All of these samples, on hematoxylin and eosin staining under light microscopy, revealed an absence of ganglion cells. A few small nerve fibers were seen but they were not prominent or hyperplastic unlike most cases of HD (Fig. 2).

**Fig. 3:**
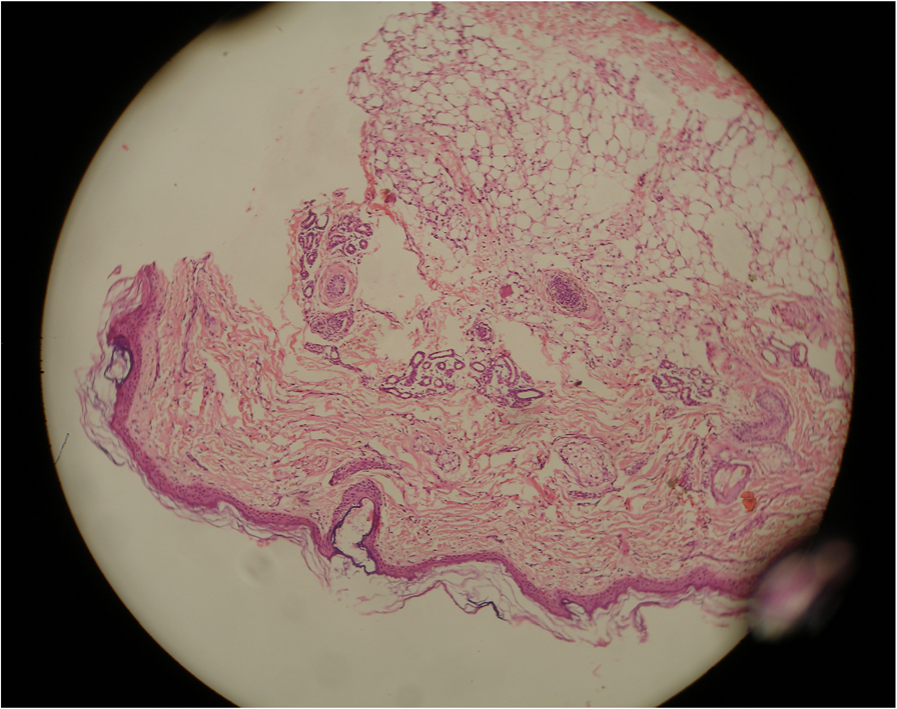
Skin biopsy from the scalp revealed normal epidermis with scanty hair follicles with no hair shafts and absence of inflammatory infiltrates

A skin biopsy from her scalp revealed scanty hair follicles with no hair shafts in the epidermal layers and no inflammatory infiltrates (Fig. 3), which was confirmative of alopecia universalis. Postoperatively, she developed enterocolitis, sepsis, and non-functional colostomy and stoma; later she died due to multiorgan failure. Her clinical features and biopsy reports confirmed our diagnosis of HD with ALUNC.

Her elder female sibling had a similar history of total absence of hair on the body since birth in association with esophageal atresia; she died on the 12th day of life. There was no history of any external congenital anomalies or deformities in the sibling and no other family member was affected by alopecia. In our case study, the occurrence of the disorder in two siblings with unaffected parents suggested an autosomal recessive mode of inheritance. Our patient’s parents were counseled for genetic testing; however, they deferred it. The mother gave birth to a third female child who had no alopecia or any congenital anomaly and the baby is now 2-months old.

## Discussion

HD is a developmental disorder with multiple genetic causes and etiologic mechanisms. Mutations in a variety of genes may be responsible for its heterogenous nature. The most common gene identified is the *RET* proto-oncogene. *RET* abnormalities are usually encountered in familial and long segment disease. Other genes associated with HD are *endothelin-3*, *endothelin-B*, *EDNRB*, *SOX-10*, *S1P1*, and *Phox2B* [4]. HD occurs as an isolated phenotype in 70 % cases and in 5 to 32 % it is associated with anomalies or syndromes. Definite associations of HD with Down’s syndrome, dominant sensorineural deafness, Waardenburg syndrome, neurofibromatosis, neuroblastoma, and the multiple endocrine neoplasia (MEN) type IIB syndrome are known. Anomalies of the gastrointestinal tract (GIT), central nervous system, sensorineural anomalies, and cardiovascular system are often associated with HD. Less frequently there could be craniofacial defects, eye abnormalities, and skin and integumentary system (ectodermal dysplasia; ED) involvement [5].

Alopecia or hypotrichosis presenting from birth includes several different forms of hereditary hair loss in humans. Congenital alopecia usually presents at or soon after birth as a localized form or generalized loss of hair (generalized atrichia). This rare genetic entity is seen either in isolation or with associated defects [3]. A few well-known hypotrichotic syndromes such as atrichia with papular lesions, GAPO syndrome (growth retardation, alopecia, pseudoanodontia, and optic atrophy), congenital atrichia with nevus flammeus, hypotrichosis with amino acid metabolism alterations, dominant hidrotic ED, as well as X-linked hypohidrotic ED, represent a broad spectrum of hereditary alopecia in children, but the association of these syndromes with HD or other gastrointestinal disorders is yet to be studied further [6].

The isolated forms of congenital alopecia are either familial or sporadic. The majority of familial cases have an autosomal recessive inheritance, although a few cases of autosomal dominant or X-linked recessive inheritance have also been reported [3]. Patients with an autosomal dominant type of congenital alopecia usually present with a less severe form with partial hair loss, which manifests in childhood or later. In contrast, the recessive form of congenital alopecia manifests as complete loss of hair development, which affects the entire scalp and body including eyebrows, eyelashes, axillary, and pubic regions. Rarely, some affected newborns have a few sparse hairs on their head, which fall off within days and these never re-grow. Classically, a skin biopsy in such individuals reveals a normal epidermis and dermis containing either hair follicles without hair or absence of hair follicles [3, 6].

Congenital alopecia and HD have both been associated with EDs. EDs include a wide range of complex pathological and clinical conditions in which common anomalies of the hair, teeth, nails, and sweat glands are seen. These may also be associated with anomalies in other organs. Three different cell types originate from the embryonic ectoderm: outer ectoderm, neural tubes, and neural crest (NC). The outer ectoderm or surface ectoderm forms epidermal layers of skin during the 5th to 11th week of fetal development. A few of the NC cells migrate and invade the epidermis and differentiate into melanocytes, which cause skin pigmentation [7]. Our patient only had alopecia; she had no abnormality of nails, skin texture, or pigmentation.

The neurons of the enteric nervous system are derived from NC cells. They begin to appear and to migrate craniocaudally at the 5th week of gestation, extending from the esophagus down to the anal canal by the 12th to 16th week. The absence of ganglion cells in HD has been attributed to arrested migration of NC cells. The length of an aganglionic segment is inversely proportional to gestational age at the onset of migration arrest [8].

In the literature, a few uncommon associations between HD and ED have been reported [5, 9, 10]. IFAP (ichthyosis follicularis with atrichia and photophobia)/BRESEK (brain anomalies, intellectual disability, ED, skeletal deformities, ear or eye anomalies, and kidney dysplasia/hypoplasia) syndrome and cartilage–hair hypoplasia (CHH) are such examples where besides other clinical features, congenital hypotrichosis and HD association have been observed [11, 12]. IFAP syndrome is a rare X-linked disorder of multiple congenital malformations manifesting often at birth with ichthyotic lesions, atrichia, short stature, and seizures [13]. Patients with BRESEK/BRESHECK syndrome present with brain anomalies, intellectual disability, ED, skeletal deformities, ear or eye anomalies, cleft palate, cryptorchidism, and renal anomalies with or without HD [10, 11]. The skin texture of our patient was normal and she had no external ear/eye anomalies, skeletal deformities, or cerebral or renal malformations.

CHH is a metaphyseal chondrodysplasia of McKusick type. In the majority of cases it is an autosomal recessive disorder; it frequently involves the skeletal system (short stature) and joint laxity together with variable features such as blond fine sparse hair and defective cellular immunity. GIT dysfunction such as malabsorption, HD, anal stenosis, and esophageal atresia are frequently observed in CHH, of which HD is the most common disorder encountered. Shortening of tubular bones with widened, scalloped, and sclerotic metaphyses is seen radiographically [14, 15]. Although frequent association between CHH and HD has been observed, in our case, prominent features of CHH such as joint hyperlaxity or skeletal changes were not seen.

Surgery is the mainstay of treatment for HD. Short-segment HD can be managed with the definitive ileoanal pull-through anastomosis. If the child has developed enterocolitis or has a significantly dilated colon, as in our case, a colostomy is required to be placed for an initial period while the child recovers; the pull-through procedure is performed 4 to 6 months after colostomy placement [1].

Treatment of cases of alopecia universalis is targeted toward protecting skin from sun burn, infection, and injuries. Prenatal counseling and genetic studies are an integral part of the management of these cases.

## Conclusions

All cases of HD should be thoroughly investigated for associations with syndromes and anomalies. Alopecia is an unusual association with HD. Congenital alopecia usually poses a diagnostic dilemma to the treating physicians and early diagnosis will help in appropriate management of these cases. Genetic evaluation of the affected individuals with HD and alopecia universalis is essential for better understanding of the disease.
